# Digital literacy is associated with digital teaching self-efficacy among pre-service physical education teachers: the indirect roles of professional identity and teaching self-efficacy

**DOI:** 10.3389/fpsyg.2026.1886664

**Published:** 2026-08-26

**Authors:** Shuang Zhao, Guoqiang Song, Ying Xiang, Peiyuan Tian, Xu Zhou, Ruoyu Zhang, Xitang Zhao

**Affiliations:** 1 School of Physical Education, Hanjiang Normal University, Shiyan, Hubei, China; 2 Physical Education Department, Rattana Bundit University, Bangkok, Thailand; 3 Nanjing Sport Institute, Nanjing, Jiangsu, China

**Keywords:** digital literacy, digital teaching self-efficacy, pre-service physical education teacher, professional identity, serial indirect pathway, teaching self-efficacy

## Abstract

**Background:**

The digital transformation of education poses new demands on physical education (PE) teachers' professional competencies. Pre-service PE teachers, as the future workforce, may have their digital teaching self-efficacy associated with digital literacy through indirect pathways.

**Methods:**

Using convenience cluster sampling, 776 pre-service PE teachers from first- to fourth-year undergraduate PE programmes in five normal universities in Hubei Province, China, were surveyed online. Participants completed measures of digital literacy, professional identity, teaching self-efficacy, and Digital Teaching Self-Efficacy. SPSS 26.0 and AMOS 31.0 were used for descriptive statistics, correlation analysis, common method bias testing, and confirmatory factor analysis. PROCESS macro (Model 6) with 5,000 bootstrap samples was employed to test the serial indirect pathway model.

**Results:**

Digital literacy was significantly and positively correlated with professional identity (*r* = 0.41), teaching self-efficacy (*r* = 0.38), and Digital Teaching Self-Efficacy (*r* = 0.44; all *p* < 0.001). The direct effect of digital literacy on Digital Teaching Self-Efficacy was significant (*b* = 0.249, β = 0.263, *p* < 0.001). The unstandardized indirect effects via professional identity and teaching self-efficacy were 0.0651 and 0.0703, respectively. The serial indirect pathway was significant, with an unstandardized effect of 0.0282, 95% CI [0.0180, 0.0409].

**Conclusion:**

This study explored the link between digital literacy and Digital Teaching Self-Efficacy among pre-service physical education teachers. The results are consistent with the model in which digital literacy is associated with digital teaching self-efficacy directly, and indirectly through professional identity, teaching self-efficacy, and their serial indirect pathway. These findings are consistent with the proposed indirect pathway linking digital literacy to higher Digital Teaching Self-Efficacy. These findings may help generate intervention hypotheses related to strengthening professional identity and providing guided digital teaching experiences. Such hypotheses should be tested through longitudinal studies or experimental designs before being translated into practice. Overall, this study offers empirical insights that may inform PE teacher preparation by integrating digital training with identity-building and self-efficacy development. Due to the cross-sectional design, all findings should be interpreted as correlational, not causal.

## 1 Introduction

Digital technologies are profoundly reshaping education. UNESCO's “Quality Physical Education” guidelines stress that PE teachers must integrate digital tools into their teaching (Uhlenbrock and Meier, 2021). Similarly, China's Education Informatization 2.0 Action Plan and the Teacher Digital Literacy industry standard highlight digital literacy as central to modernizing education (Yan and Yang, 2021).

Pre-service PE teachers are the future reserve force of school physical education; their level of digital literacy is closely linked to their own professional development and with students' motor skill acquisition (Zhao et al., 2025). However, whether mere operational skills with digital technology are sufficient for pre-service PE teachers to demonstrate high-quality teaching performance remains to be explored (Nadal et al., 2026; Zhang et al., 2025). Professional identity and teaching self-efficacy may play important mediating roles in this association (Cai et al., 2022).

Social cognitive theory (Bandura, 2001) and self-determination theory (Sun et al., 2017) provide a theoretical foundation for understanding how digital literacy relates to Digital Teaching Self-Efficacy. The former emphasizes the interplay between external environmental factors and self-beliefs, while the latter focuses on the role of role-identity in autonomous behavior (Yi et al., 2025). In addition, conservation of resources (COR) theory (Hobfoll, 2001) suggests that individuals strive to obtain and protect resources, and that resource gains may contribute to further resource accumulation. From this perspective, digital literacy can be viewed as an external resource that may enable pre-service teachers to develop professional identity as psychological capital, which may foster teaching self-efficacy and contribute to Digital Teaching Self-Efficacy. Digital Teaching Self-Efficacy refers to individuals' beliefs in their capability to effectively perform digital-technology-integrated teaching tasks. According to social cognitive theory, such beliefs are shaped by mastery experiences, vicarious experiences, and social persuasion, rather than reflecting objective competence *per se* (Bandura, 2001). For pre-service PE teachers, capability in this context encompasses beliefs about managing technology-enhanced classrooms, demonstrating digitally supported motor skills, and providing individualized instruction using digital tools (Marinsek and Kovac, 2018). Previous research has shown that digital literacy tends to co-occur with stronger instructional design, resource development and evaluation abilities (Scherer et al., 2019). Yet PE teaching is characterized by high physical load and real-time interaction; the specific way in which digital literacy connects to pre-service PE teachers' Digital Teaching Self-Efficacy remains under-explored (Gustian et al., 2026; Østerlie et al., 2025). Accordingly, we propose the following hypotheses:

H1: digital literacy is positively associated with Digital Teaching Self-Efficacy among pre-service PE teachers.

Professional identity refers to an individual's cognitive and emotional perception of the value, meaning and sense of belonging to the teacher role (Anwar et al., 2025; Sönmez et al., 2026). According to social cognitive theory, external environmental factors, such as mastering digital technology, influence self-beliefs and values, which in turn shape behavioral engagement (Bandura, 2001). When pre-service PE teachers possess high digital literacy, they are more likely to gain a sense of accomplishment from using digital tools, and this positive experience tends to reinforce their professional identification with the PE teacher role (Wang and Ye, 2026; Zhang et al., 2024). Moreover, teachers with stronger professional identity invest more effort in improving their teaching ability, and such effort parallels higher Digital Teaching Self-Efficacy (Liu and Keating, 2022; Yang et al., 2023) Therefore, we propose:

H2: professional identity mediates the association between digital literacy and Digital Teaching Self-Efficacy among pre-service PE teachers.

Teaching self-efficacy refers to teachers' belief in their ability to effectively perform teaching tasks and promote student learning (Cruz et al., 2020). Pre-service PE teachers with higher digital literacy tend to handle technical aspects more confidently, and such confidence accompanies higher self-efficacy (Ma et al., 2026; Zeng et al., 2022). A meta-analysis has shown that teacher self-efficacy is positively linked to teaching behavior and student outcomes (Zee and Koomen, 2016). Among pre-service PE teachers, those with higher self-efficacy are more willing to adopt innovative teaching methods, manage classrooms effectively, and thus report higher Digital Teaching Self-Efficacy (Semiz and Ince, 2012; Yu and Alibakhshi, 2025; Zhao et al., 2025). Thus, we propose:

H3: teaching self-efficacy mediates the association between digital literacy and Digital Teaching Self-Efficacy among pre-service PE teachers.

Professional identity and teaching self-efficacy may have a serial associative chain (Cai et al., 2022). Self-determination theory states that when individuals identify with a role, they autonomously engage in related activities and develop corresponding competence beliefs, meaning that identity fosters self-efficacy beliefs (Gorozidis et al., 2020; Sun et al., 2017). For pre-service PE teachers, the stronger their identification with the PE teacher profession, the more actively they seek professional growth and accumulate successful teaching experiences, which in turn contributes to higher teaching self-efficacy (González-Calvo and Fernández-Balboa, 2018; Huang et al., 2025; Sen, 2023). Based on this, we propose:

H4: professional identity and teaching self-efficacy serially mediate the association between digital literacy and Digital Teaching Self-Efficacy among pre-service PE teachers.

**Figure 1:**
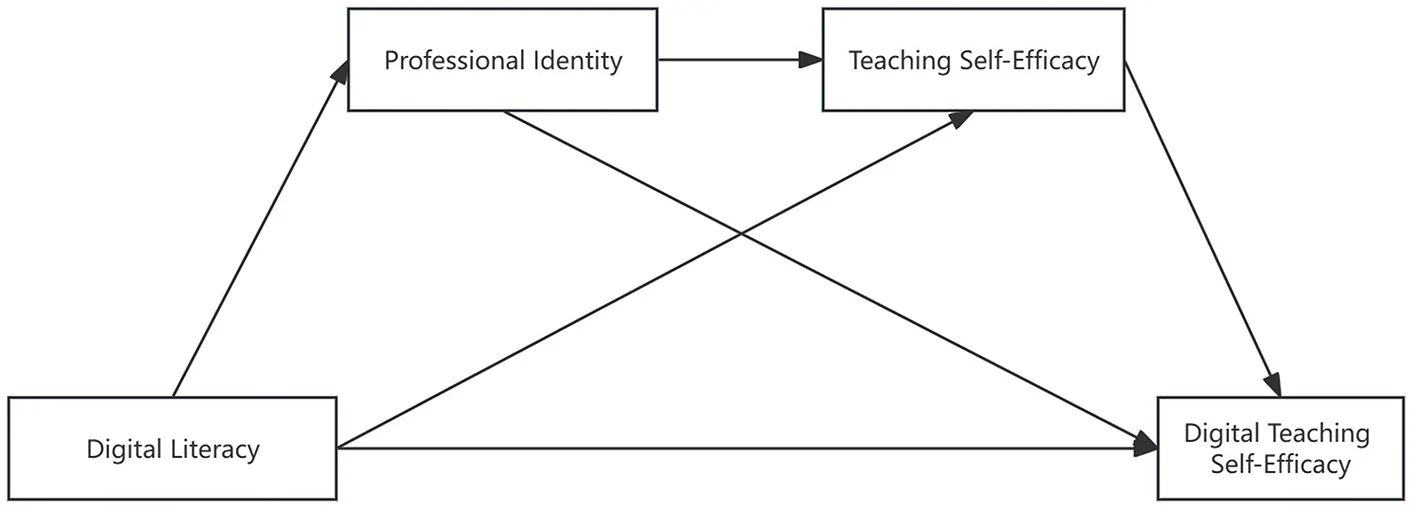
Hypothesized serial indirect pathway model.

To test these hypotheses, we constructed a serial indirect pathway model (Figure 1). The model is grounded in theoretical frameworks and serves as a plausible representation of the hypothesized associations, rather than a test of causal ordering.

## 2 Methods

### 2.1 Participants and procedure

A convenience sampling method was adopted. First- to fourth-year undergraduate physical education majors were recruited from five normal universities in Hubei Province, China, with whole classes selected based on accessibility. Data were collected between May 12, 2025 and January 31, 2026. The questionnaire was administered online via the Wenjuanxing platform (a Chinese online survey platform). A total of 800 responses were collected; after excluding incomplete or careless responses (defined as missing responses on more than three items, straight-lining across ≥80% of items, or completion time below three standard deviations of the mean), 776 valid questionnaires were retained, yielding a valid response rate of 97.0%. This high retention rate is primarily attributable to the use of an online survey platform with forced-response validation, which minimized missing data, and the relatively short completion time (approximately 10–15 min), which reduced participant fatigue and dropout. The sample comprised 412 males and 364 females, with 234 freshmen, 218 sophomores, 186 juniors, and 138 seniors (aged 18–24 years; *M* = 20.34, SD = 1.27). Participants provided electronic informed consent by clicking a consent button before proceeding. Consent was not obtained in writing, as the survey was conducted entirely online. This study was reviewed and approved by the Medical Ethics Committee of Wuhan University (Approval No. WHU-LFMD-IRB2025039). Detailed demographic characteristics are presented in Table 1.

**Table 1:** Sample demographic characteristics (*N* = 776).

Characteristics	Category	Sample size	Percentage
Gender	Male	412	53.09%
	Female	364	46.91%
Grade	Freshman	234	30.15%
	Sophomore	218	28.09%
	Junior	186	23.97%
	Senior	138	17.78%
Age	18 years	52	6.70%
	19 years	118	15.21%
	20 years	182	23.45%
	21 years	196	25.26%
	22 years	136	17.53%
	23 years	92	11.86%
Internship experience	Yes	271	34.92%
	No	505	65.08%
Digital training	Participated	367	47.29%
	Not participated	409	52.71%
Exercise habit	Never	156	20.10%
	Occasionally	348	44.85%
	Regularly	272	35.05%

Due to the fully anonymised design of the online survey, the study did not retain class identifiers. All 776 observations were therefore treated as independent in the primary analyses. However, university affiliation was recorded for each participant, allowing us to create dummy-coded institutional covariates to adjust for potential between-university heterogeneity in sensitivity analyses.

### 2.2 Sample size estimation

An *a priori* power analysis was performed using G^*^Power 3.1.9.2 (Faul et al., 2007) for the regression paths in our model. For a linear multiple regression model with six predictors (digital literacy, professional identity, teaching self-efficacy, gender, grade, and internship experience), a medium effect size (*f* ^2^ = 0.15), α = 0.05 (two-tailed), and desired power 1–β = 0.80, the minimum required sample size was estimated to be 98. Our final sample of 776 participants substantially exceeds this number, indicating sufficient power for the regression components of the model.

Given that testing the indirect effects within a serial indirect pathway model involves the product of multiple regression paths, we also evaluated sample size adequacy using recommendations specific to mediation analysis. According to (Fritz and MacKinnon, 2007), a sample size of 300–500 is generally sufficient to detect small-to-medium indirect effects with adequate power (≥ 0.80) in mediation models. (Schoemann et al., 2017) further recommend that complex serial indirect pathway models with moderate effect sizes require approximately 300–500 participants to achieve stable estimates. Our sample of 776 participants exceeds these benchmarks, confirming sufficient statistical power to detect the hypothesized indirect effects.

To further evaluate the minimum sample size required to achieve adequate power, a series of simulation-based *post hoc* sensitivity analyses were conducted using Mplus 8.3 with 1,000 replications per condition, varying sample sizes from 100 to 300 based on the observed path coefficients from the sample. The results showed that the serial indirect effect achieved statistical power of 0.236 at *N* = 100, 0.849 at *N* = 200, and approached 1.000 at *N* = 300. These findings indicate that a sample size of approximately 200 participants is sufficient to achieve 0.80 power for detecting the serial indirect effect. The actual sample (*N* = 776) substantially exceeds this threshold. As these analyses relied on empirical path coefficients derived from the data, they represent *post hoc* sensitivity checks rather than a standalone *a priori* power analysis. Supplementary File S11 contains the complete Mplus syntax and all parameter specifications, including path coefficients, residual variances, distributional assumptions, and indirect effect parameters.

### 2.3 Measures

All scales were originally developed in English. To ensure cultural and linguistic appropriateness for the Chinese context, the scales were translated into Chinese by two bilingual researchers independently. The translated versions were then back-translated into English by two independent translators who were blinded to the original versions. Discrepancies were resolved through discussion among the research team. A pilot test with 30 pre-service PE teachers was conducted to assess clarity and comprehension, and no substantial modifications were needed. To mitigate potential common method bias, different scale anchors (5-point, 7-point, and 9-point) were used across the four measures.

#### 2.3.1 Digital literacy scale

The Digital Literacy Scale was adapted from (Ng, 2012). It contains 10 items measuring digital tool operation, information evaluation, and digital content creation. All items were rated on a 5-point Likert scale, ranging from 1 (strongly disagree) to 5 (strongly agree). In this study, the scale demonstrated good internal consistency, with a Cronbach's alpha of 0.926.

#### 2.3.2 Professional identity scale

The Professional Identity Scale was developed by (Liu and Keating, 2022) for pre-service PE teachers. It comprises 17 items covering professional value, professional affect, and professional behavioral tendencies. Responses were provided on a 7-point Likert scale, with 1 representing strongly disagree and 7 representing strongly agree. One reverse-coded item was reverse-scored before computing the mean score. The scale demonstrated high internal consistency in this sample, with a Cronbach's alpha of 0.92.

#### 2.3.3 Teaching self-efficacy scale

The short form of the Teachers' Sense of Efficacy Scale (Tschannen-Moran and Hoy, 2001) was used. It comprises 12 items across three dimensions: efficacy for classroom management, student engagement, and instructional strategies. Items are rated on a 9-point scale (1 = nothing, 9 = a great deal), with a Cronbach's alpha of 0.91.

#### 2.3.4 Digital teaching self-efficacy scale

Digital Teaching Self-Efficacy was assessed using the teaching execution subscale of the Scale of Competency of Digital Age Teaching (Kang et al., 2024). This subscale measures preservice teachers' capability to execute digital technology-integrated instruction in authentic classroom settings. It consists of 6 items, all rated on a 5-point Likert scale ranging from 1 (strongly disagree) to 5 (strongly agree). The scale was translated into Chinese following the procedure described at the beginning of Section 2.3. In this study, the subscale demonstrated satisfactory internal consistency (Cronbach's α = 0.88). The full questionnaire items for all scales are provided in Supplementary Table S8.

### 2.4 Data analysis

Confirmatory factor analysis (CFA) was performed using AMOS 31.0 with maximum likelihood estimation. Descriptive statistics, correlation analysis, and common method bias testing were performed using IBM SPSS Statistics 26.0. Gender, grade, and internship experience were included as control variables to account for potential demographic and experiential differences (coded as follows: Gender: 1 = male, 2 = female; Grade: 1 = freshman, 2 = sophomore, 3 = junior, 4 = senior; Internship: 1 = yes, 2 = no). All continuous predictors were mean-centered prior to analysis. No heteroscedasticity-consistent standard errors were used; standard OLS standard errors are reported. A random seed of 12345 was set for bootstrap sampling to ensure reproducibility. The proportion of the total effect mediated was calculated as the indirect effect divided by the total effect (Preacher and Hayes, 2004). A serial indirect pathway model was tested using PROCESS macro for SPSS Version 4.2 (Model 6) (Hayes, 2022), with 5,000 bootstrap samples to generate bias-corrected 95% confidence intervals for the indirect effects. To further address potential clustering at the university level, we created four dummy variables for university affiliation (with one institution as the reference category) and entered them as fixed-effect covariates in all regression equations of the serial indirect pathway model (PROCESS Model 6). Supplementary regression diagnostics, including residual plots, VIFs, and Durbin-Watson statistics, are provided in Supplementary Figures S1–3.

## 3 Results

### 3.1 Common method bias test

Harman's single-factor test was performed on all items of the four scales (digital literacy, professional identity, teaching self-efficacy, and Digital Teaching Self-Efficacy). An unrotated exploratory factor analysis extracted four factors with eigenvalues greater than 1. The first factor accounted for 32.96% of the total variance, below the 40% critical threshold (Podsakoff et al., 2003).

### 3.2 Descriptive statistics and correlation analysis

Table 2 presents the means, standard deviations, and Pearson correlations among the study variables. Digital literacy was significantly and positively correlated with professional identity (*r* = 0.413, *p* < 0.001), teaching self-efficacy (*r* = 0.384, *p*<*0.001*), and Digital Teaching Self-Efficacy (*r* = 0.435, *p* < 0.001). Professional identity was positively correlated with teaching self-efficacy (*r* = 0.379, *p* < 0.001) and Digital Teaching Self-Efficacy (*r* = 0.380, *p* < 0.001). Teaching self-efficacy was positively correlated with Digital Teaching Self-Efficacy (*r* = 0.434, *p* < 0.001). The correlation matrix supported further mediation analysis.

**Table 2:** Descriptive statistics and pearson correlation matrix.

Variable	Mean	SD	1	2	3	4
1. Digital literacy	3.735	0.856	1			
2. Professional identity	4.319	1.251	0.413^***^	1		
3. Teaching self-efficacy	6.357	1.481	0.384^***^	0.379^***^	1	
4. Digital teaching self-efficacy	3.914	0.812	0.435^***^	0.380^***^	0.434^***^	1

^***^p < 0.001.

To address potential overlap between teaching self-efficacy (TSE) and Digital Teaching Self-Efficacy (DTSE) measures, a detailed content analysis of the items was conducted. The TSE scale (α = 0.91) focuses on participants' confidence in their ability to perform specific teaching tasks, while the DTSE scale (α = 0.88) assesses participants' perceptions of their overall digital teaching self-efficacy across multiple domains. A confirmatory factor analysis (CFA) with two distinct factors (TSE and DTSE) showed good model fit (χ^2^/df = 1.319, CFI = 0.995, TLI = 0.994, RMSEA = 0.020, RMR = 0.057), and the correlation between the two factors was 0.475. The HTMT value for these two constructs was 0.477, which is well below the conventional 0.85 threshold, indicating good discriminant validity. These results indicate that TSE and DTSE are distinguishable constructs, while acknowledging their conceptual proximity.

### 3.3 Confirmatory factor analysis

To examine the construct validity of the scales, confirmatory factor analysis (CFA) was conducted on digital literacy, professional identity, teaching self-efficacy, and Digital Teaching Self-Efficacy. The model fit indices are presented in Table 3. The results indicated a good fit: χ^2^/*df* = 1.376, CFI = 0.983, TLI = 0.982, RMSEA = 0.022, SRMR = 0.028.

**Table 3:** Confirmatory factor analysis: model fit indices.

χ^2^	df	*p*	χ^2^/df	CFI	TLI	RMSEA	SRMR
1,292.445	939	0.000	1.376	0.983	0.982	0.022	0.028

Good fit is indicated by χ^2^/df < 3, CFI/TLI >0.9, RMSEA < 0.08, SRMR < 0.08.

Table 4 presents the average variance extracted (AVE) and composite reliability (CR) for each latent variable. The AVE for each latent variable exceeded 0.5, and CR exceeded 0.7, demonstrating adequate convergent validity and internal consistency. All factor loadings were significant at *p* < 0.001 and exceeded 0.65.

**Table 4:** Average variance extracted and composite reliability.

Latent variable	Average variance extracted	Composite reliability
Digital literacy	0.560	0.926
Professional identity	0.509	0.946
Teaching self-efficacy	0.566	0.940
Digital teaching self-efficacy	0.558	0.883

Average variance extracted >0.5 indicates acceptable convergent validity; composite reliability >0.7 indicates good internal consistency. All factor loadings are significant at p < 0.001 and exceed 0.65.

### 3.4 Serial indirect pathway effect test

Using PROCESS macro (Model 6) with digital literacy as the independent variable, Digital Teaching Self-Efficacy as the dependent variable, professional identity and teaching self-efficacy as serial mediators, and controlling for gender, grade, and internship experience, the regression coefficients for each path are presented in Table 5.

**Table 5:** Regression results for the serial indirect pathway model.

Outcome variable	Predictor	*b*	*SE*	β	*t*	*p*	*R^2^*
Professional identity	Digital literacy	0.604	0.048	0.413	12.575	< 0.001	0.171
Teaching self-efficacy	Digital literacy	0.472	0.061	0.273	7.723	< 0.001	0.208
	Professional identity	0.315	0.042	0.266	7.563	< 0.001	
Digital teaching self-efficacy	Digital literacy	0.249	0.033	0.263	7.609	< 0.001	0.296
	Professional identity	0.110	0.022	0.170	4.936	< 0.001	
	Teaching self-efficacy	0.148	0.019	0.270	7.950	< 0.001	

b, unstandardized coefficient; SE, standard error; β, standardized coefficient. Control variables (gender, grade, and internship) were included in all models. Complete regression coefficients for all variables, including control variables, are presented in Supplementary Table S12. In the present model, none of the control variables reached statistical significance (all p > 0.05).

The results showed that digital literacy was positively associated with professional identity (*b* = 0.604, *SE* = 0.048, β = 0.413, *p* < 0.001) and teaching self-efficacy (*b* = 0.472, *SE* = 0.061, β = 0.273, *p* < 0.001). Professional identity was positively associated with teaching self-efficacy (*b* = 0.315, SE = 0.042, β = 0.266, *p* < 0.001) and Digital Teaching Self-Efficacy (*b* = 0.110, *SE* = 0.022, β = 0.170, *p* < 0.001). Teaching self-efficacy was positively associated with Digital Teaching Self-Efficacy (*b* = 0.148, SE = 0.019, β = 0.270, *p* < 0.001). The direct effect of digital literacy on Digital Teaching Self-Efficacy was significant (*b* = 0.249, *SE* = 0.033, β = 0.263, *p* < 0.001). The *R*^2^ values for the three outcome variables were 0.171, 0.208, and 0.296, respectively.

The focal regression coefficients are presented in Table 5. Complete coefficients for all variables, including gender, grade, and internship experience, are reported in Supplementary Table S12. In the present model, none of the control variables reached statistical significance (all *p* > 0.05).

**Figure 2:**
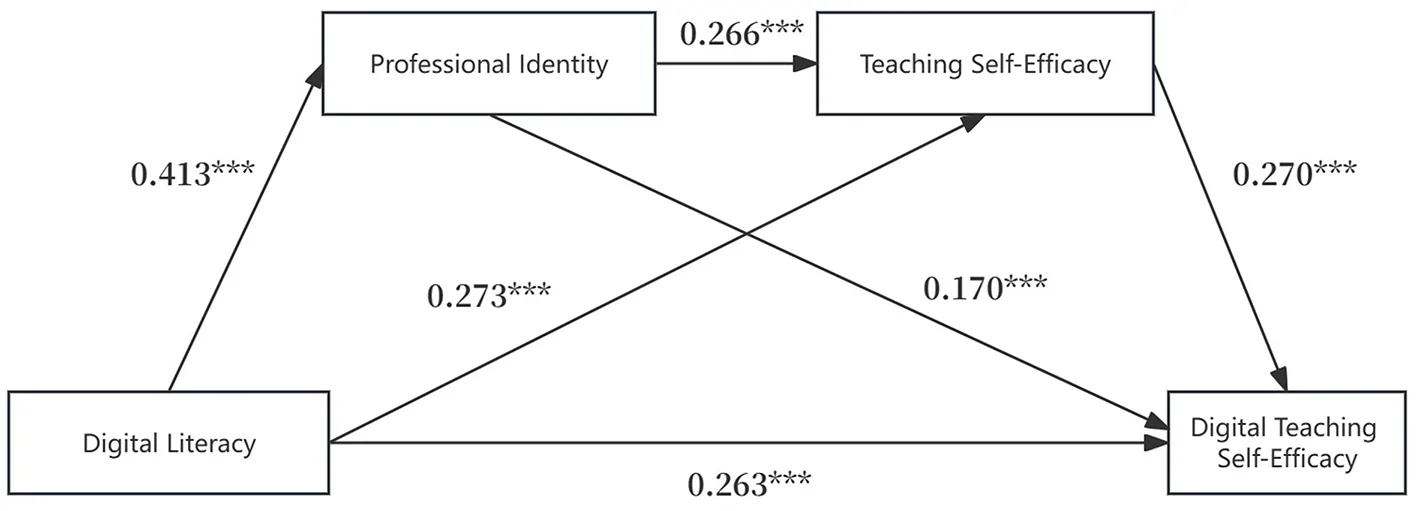
Chain mediation model diagram with standardized path coefficients. Path coefficients are standardized. Control variables (gender, grade, internship experience) were included in the model but are not shown for clarity. *p* < 0.001. *N* = 776. Sensitivity analyses with university affiliation as fixed-effect covariates yielded virtually identical results. The symbol ^***^ indicates *p* < 0.001, denoting that the path coefficient is statistically highly significant.

To visualize the relationships among variables, a path diagram was drawn (Figure 2).

### 3.5 Bootstrap indirect effects

Bootstrap results (Table 6) with 5,000 resamples (Preacher and Hayes, 2004) showed that the total unstandardized indirect effect was 0.1640 (95% CI [0.1281, 0.2001]), accounting for 39.8% of the total effect. All three indirect paths were significant:

- Path 1 (digital literacy → professional identity → digital teaching self-efficacy): effect = 0.0651, 95% CI [0.0373, 0.0940].
- Path 2 (digital literacy → teaching self-efficacy → digital teaching self-efficacy): effect = 0.0703, 95% CI [0.0468, 0.0969].
- Path 3 (digital literacy → professional identity → teaching self-efficacy → digital teaching self-efficacy): effect = 0.0282, 95% CI [0.0180, 0.0409].

**Table 6:** Indirect effects of the serial indirect pathway model.

Type of effect	Effect size	95% bootstrap CI		Proportion of total effect
		LLCI	ULCI	
Total effect	0.4141	0.3536	0.4746	—
Direct effect	0.2494	0.1851	0.3138	60.2%
Total indirect effect	0.1640	0.1281	0.2001	39.8%
Indirect effect 1	0.0651	0.0373	0.0940	16.1%
Indirect effect 2	0.0703	0.0468	0.0969	16.9%
Indirect effect 3	0.0282	0.0180	0.0409	6.8%

Indirect effect 1: digital literacy → professional identity → digital teaching self-efficacy; Indirect effect 2: digital Literacy → teaching self-efficacy → digital teaching self-efficacy; Indirect effect 3: digital Literacy → professional identity → teaching self-efficacy → digital teaching self-efficacy. All total, direct, and indirect effect values presented in Table 6 are unstandardized. Standardized serial indirect effect comparisons for sensitivity analysis are reported in Section 3.6. Bootstrap resamples = 5,000. 95% confidence intervals that do not contain zero indicate statistical significance.

The serial indirect pathway estimate accounted for 6.8% of the total association, and the confidence interval did not contain zero. These findings are consistent with Hypothesis 4.

### 3.6 Sensitivity analyses

To examine the robustness of the serial indirect pathway model, three supplementary analyses were conducted: (a) a parallel mediation model (PROCESS Model 4) with professional identity (PI) and teaching self-efficacy (TSE) as simultaneous mediators, (*b*) a reverse-ordered serial indirect pathway model (PROCESS Model 6) specifying TSE as the first mediator and PI as the second mediator (DL → TSE → PI → DTSE), and (c) a sensitivity analysis treating university affiliation as fixed-effect covariates. All effects in this section are reported as standardized estimates. These are presented for comparison purposes only and should not be directly compared with the unstandardized effects reported in Table 6.

The parallel mediation model showed that both indirect effects were significant: DL → TSE → DTSE (standardized effect = 0.1034, 95% CI [0.0756, 0.1356]) and DL → PI → DTSE (standardized effect = 0.0703, 95% CI [0.0408, 0.1010]). The contrast between the two indirect paths was not significant (C1 = 0.0332, 95% CI [−0.0136, 0.0806]), indicating comparable indirect effects through both mediators.

Reverse-ordered serial indirect pathway model. When the order of mediators was reversed (DL → TSE → PI → DTSE), the serial indirect effect was also significant but notably smaller (standardized effect = 0.0169, 95% CI [0.0092, 0.0262]) compared to the proposed model (standardized effect = 0.0282, 95% CI [0.0180, 0.0402]). The contrast between the indirect effect via professional identity alone (Ind1: DL → PI → DTSE) and the serial indirect effect (Ind3: DL → PI → TSE → DTSE) was statistically significant (C3 = 0.0421, 95% CI [0.0185, 0.0679]), indicating that the serial indirect pathway accounts for more indirect variance than the single-mediator pathway via professional identity. Nevertheless, because the data are cross-sectional, no conclusions about temporal precedence can be drawn. Both models are statistically plausible, and the proposed order should be interpreted as theoretically coherent rather than causally established.

To assess the assumptions underlying the regression analyses, residual plots and collinearity statistics were examined. Variance inflation factors (VIFs) ranged from 1.004 to 1.305, indicating no multicollinearity concerns. Visual inspection of residual plots supported the assumptions of linearity, homoscedasticity, and approximate normality (see Supplementary Figures S1–3). For the university-level clustering sensitivity analysis, we re-estimated the serial indirect pathway model with four university dummy variables added as fixed-effect covariates. The serial indirect effect remained statistically significant (*b* = 0.0286, 95% CI [0.0180, 0.0409]), closely replicating the main analysis without university covariates (*b* = 0.0282, 95% CI [0.0180, 0.0402]). The adjusted estimate from this sensitivity analysis is 0.0286, whereas the primary estimate remains 0.0282; both estimates are reported for transparency.

## 4 Discussion

This is an exploratory, cross-sectional study that examines statistical associations rather than causal mechanisms. The results were consistent with all four hypotheses: digital literacy was positively associated with Digital Teaching Self-Efficacy; professional identity functioned as an independent indirect correlate consistent with the hypothesized mediation pathway; teaching self-efficacy operated as a further independent indirect correlate consistent with the hypothesized mediation pathway; and professional identity together with teaching self-efficacy exhibited a serial indirect pathway between digital literacy and Digital Teaching Self-Efficacy. These findings integrate social cognitive theory (Bandura, 2001) and self-determination theory (Deci et al., 2017), offering a plausible framework for understanding how digital literacy is associated with Digital Teaching Self-Efficacy. Due to the cross-sectional design, all indirect effects should be interpreted as statistical associations rather than causal processes.

### 4.1 Direct association between digital literacy and digital teaching self-efficacy

Digital literacy was positively associated with Digital Teaching Self-Efficacy among pre-service PE teachers. This result is consistent with the meta-analytic conclusion of (Scherer et al., 2019) that teachers' ICT competence is positively correlated with teaching quality. From the perspective of social cognitive theory, teachers with higher digital literacy tend to use data analysis more effectively to inform instructional decisions (Aslan et al., 2025; De León et al., 2023). In the PE context, characterized by high physical load and real-time interaction, pre-service teachers with higher digital literacy reported higher Digital Teaching Self-Efficacy. This association is consistent with the theoretical expectation that beliefs about one's capability are linked to greater confidence in integrating digital tools (Gustian et al., 2026; Lapesigue, 2024). This association is not interpreted as evidence of actual teaching performance, as self-efficacy beliefs and objective competence are conceptually distinct.

It is worth noting that, although the serial indirect effect (*b* = 0.0282) and the variance explained by the full model (*R*^2^ = 0.296) may appear modest, they are comparable to effect sizes reported in similar educational and psychological mediation studies (e.g., Ma et al., 2026; Polat et al., 2025). In the context of complex indirect pathways involving multiple mediators, such effect sizes are considered meaningful, particularly given the cross-sectional nature of the data.

### 4.2 Indirect pathway via professional identity

Professional identity was consistent with an independent indirect pathway in the association between digital literacy and Digital Teaching Self-Efficacy. According to social cognitive theory, external environmental factors, such as mastering digital technology, are related to individuals' self-beliefs and values, which in turn are linked to behavioral engagement (Park and Kim, 2026). When pre-service PE teachers have higher digital literacy, they are more likely to gain a sense of accomplishment from using digital tools, and this positive experience may be linked to stronger professional identification with the PE teacher role (Wang and Ye, 2026; Zhang et al., 2024). In this study, professional identity was positively correlated with Digital Teaching Self-Efficacy (β = 0.170, *p* < 0.001), echoing the findings of (Park and Kim, 2026) and (Li et al., 2026; Polat et al., 2025) that stronger teacher identification is closely associated with greater instructional effort. This pathway is consistent with the idea that pre-service PE teachers with higher digital literacy tend to show stronger professional identity, which is in turn related to higher Digital Teaching Self-Efficacy. Thus, compared with merely teaching operational skills, helping teachers develop the belief that “technology empowers PE teaching” may be even more critical (Chróinín and O'Sullivan, 2016; Wei, 2025).

### 4.3 Indirect pathway via teaching self-efficacy

Teaching self-efficacy was consistent with an independent indirect pathway in the association between digital literacy and Digital Teaching Self-Efficacy. This finding aligns with previous studies (Amilusholihah et al., 2025), which found that digital literacy was significantly positively associated with teachers' self-efficacy (Liu, 2026). From the perspective of self-efficacy theory, pre-service PE teachers with higher digital literacy tend to handle technical aspects more confidently, and such confidence is associated with higher self-efficacy (Tabaru Örnek, 2026). In this study, teaching self-efficacy was positively correlated with Digital Teaching Self-Efficacy (β = 0.270, *p* < 0.001), which is consistent with findings from previous research (Dong and Aziz, 2024; Ma et al., 2026): higher self-efficacy often comes with greater willingness to adopt innovative teaching methods and more effective classroom management. This suggests that pre-service PE teacher training should include step-by-step digital teaching practice opportunities, allowing them to build self-efficacy through repeated successful experiences.

### 4.4 Serial indirect pathway via professional identity and teaching self-efficacy

The serial indirect pathway was statistically significant, and its magnitude was numerically larger than the serial indirect pathway estimated with reversed mediators. This pattern is consistent with the theoretically hypothesized ordering of mediators. However, because the data are cross-sectional, this consistency should not be interpreted as evidence of temporal sequencing. The proposed model is one of several plausible accounts of the observed covariance structure. The serial indirect pathway should therefore be understood as a statistically indirect relation that is coherent with the theoretical framework, rather than as evidence of a sequential psychological process. All claims about mediator ordering are theoretical and not empirically established through temporal evidence.

Drawing on self-determination theory, when individuals identify with a role, they are more likely to internalize its values and engage in related activities autonomously. Such autonomous engagement tends to satisfy their basic psychological need for competence, thereby fostering stronger self-efficacy beliefs (Deci et al., 2017). These findings are also loosely consistent with conservation of resources theory, which suggests that initial resources may contribute to the accumulation of further psychological resources (Hobfoll, 2001).

It is also worth noting that digital literacy and Digital Teaching Self-Efficacy, while conceptually distinct, may share some variance due to their mutual focus on technology use in educational contexts. However, our CFA and discriminant validity tests confirmed that the two constructs are empirically separable, with HTMT = 0.477, which is well below the conventional 0.85 threshold (see Supplementary Tables S5, 6).

Future research could extend these findings by examining analogous serial indirect pathway processes in other educational domains or with different mediating variables, such as teacher emotions or job crafting (Cai et al., 2022; Gorozidis et al., 2020; Sen, 2023). On a practical level, when designing digital literacy training, PE teacher education programmes might consider cultivating pre-service teachers' professional identity—for instance, through authentic school observations and digital case discussions that help them construct an image of a good teacher who is adept at using technology.

### 4.5 Limitations and future directions

This study has several limitations.

First, the cross-sectional design precludes causal inferences; future research could employ longitudinal or experimental designs.

Second, the sample was drawn from only five normal universities in Hubei Province, limiting generalizability to other regions, in-service teachers, or educational systems with different digital infrastructure.

Third, digital literacy was measured by self-report, which may introduce social desirability bias; future studies could incorporate behavioral data or classroom observations.

Fourth, due to the fully anonymised design, the study did not retain class identifiers, preventing the use of multilevel modeling at the class level. However, our sensitivity analysis treating university affiliation as a fixed-effect covariate yielded results virtually identical to the main analysis, suggesting that between-institution heterogeneity did not substantially influence the core findings. Nevertheless, because class-level clustering (students within the same class sharing instructors and resources) could not be directly modeled, the findings should be interpreted as exploratory and hypothesis-generating rather than confirmatory. Future studies should collect class-level identifiers for hierarchical linear modeling.

Fifth, although Harman's single-factor test indicated that common method bias was below the 40% threshold, this test has known limitations. Given the self-report, cross-sectional nature of the data, common method variance cannot be fully ruled out and may have inflated the observed associations. Thus, the findings should be interpreted with caution. Future studies could use multi-source data collection and longitudinal designs to mitigate this concern.

Sixth, other variables—such as school support or the quality of digital training—might also play moderating roles and warrant further investigation.

## 5 Conclusion

This exploratory, cross-sectional study examined statistical associations rather than causal relationships, specifically exploring the link between digital literacy and Digital Teaching Self-Efficacy among pre-service physical education teachers. The results are consistent with a model in which digital literacy is associated with digital teaching self-efficacy directly, and indirectly through professional identity, teaching self-efficacy, and their serial indirect pathway. These findings are consistent with an indirect pathway linking digital literacy to higher Digital Teaching Self-Efficacy. These findings may help generate intervention hypotheses related to strengthening professional identity and providing guided digital teaching experiences. Such hypotheses should be tested through longitudinal studies or experimental designs before being translated into practice. Overall, this study offers empirical insights that may inform PE teacher preparation by integrating digital training with identity-building and self-efficacy development.

## Data Availability

The original contributions presented in the study are included in the article/Supplementary material, further inquiries can be directed to the corresponding author.
