# Supplementary material for: Digital literacy is associated with digital teaching self-efficacy among pre-service physical education teachers: the indirect roles of professional identity and teaching self-efficacy
Source: Front Psychol. 2026 Aug 26;17:1886664. doi: 10.3389/fpsyg.2026.1886664 (PMC13553485; doi:10.3389/fpsyg.2026.1886664)
Supplement: Supplementary file 1 [file Table_2.docx]

### **Supplementary Figure S1. Normal P-P Plot of Standardized Residuals**

Note: Normal P-P plot of standardized residuals for the regression model predicting Digital Teaching Self-Efficacy. Points closely follow the diagonal line, indicating approximate normality of residuals.

**Supplementary Figure S2. Scatterplot of Standardized Residuals vs. Standardized Predicted Values**

Note: Scatterplot of standardized residuals against standardized predicted values for the regression model predicting Digital Teaching Self-Efficacy. Residuals are randomly distributed around zero with no obvious patterns, supporting the assumptions of linearity and homoscedasticity.

### **Supplementary Figure S3. Histogram of Standardized Residuals**

Note: Histogram of standardized residuals for the regression model predicting Digital Teaching Self-Efficacy. The distribution approximates normality, with a mean near zero (-1.03E-15) and a standard deviation of 0.996 (N = 776).
