# Supplementary material for: Digital literacy is associated with digital teaching self-efficacy among pre-service physical education teachers: the indirect roles of professional identity and teaching self-efficacy
Source: Front Psychol. 2026 Aug 26;17:1886664. doi: 10.3389/fpsyg.2026.1886664 (PMC13553485; doi:10.3389/fpsyg.2026.1886664)
Supplement: Supplementary file 2 [file Table_3.docx]

**Supplementary_Tables_S1-S12**

**Table S1. KMO and Bartlett’s Test of Sphericity**

| Kaiser–Meyer–Olkin (KMO) Measure | 0.971 |
| --- | --- |
| Bartlett’s Test of Sphericity | Approx. Chi-Square = 20818.749, df = 990, ***p < 0.001 |

Note: KMO > 0.8 and a significant Bartlett’s test indicate that the data are highly suitable for factor analysis.

**Table S2. Total Variance Explained**

| Factor | Eigenvalue | | | Rotated | | |
| --- | --- | --- | --- | --- | --- | --- |
|  | Eigenvalue | Explained Variance (%) | Cumulative (%) | Eigenvalue | Explained Variance (%) | Cumulative (%) |
| 1 | 14.830 | 32.956 | 32.956 | 9.155 | 20.345 | 20.345 |
| 2 | 5.112 | 11.359 | 44.315 | 7.338 | 16.307 | 36.652 |
| 3 | 3.944 | 8.765 | 53.081 | 6.094 | 13.542 | 50.194 |
| 4 | 2.400 | 5.334 | 58.415 | 3.699 | 8.221 | 58.415 |

Note: Extraction method: Principal component analysis. Rotation method: Varimax with Kaiser normalisation. The first four factors with eigenvalues > 1 accounted for 58.42% of the total variance.

**Table S3. Rotated Factor Loadings**

| Items | Factor 1 | Factor 2 | Factor 3 | Factor 4 | Communality |
| --- | --- | --- | --- | --- | --- |
| PI1 – PI17 | 0.666 – 0.785 | – | – | – | 0.482 – 0.661 |
| TSE1 – TSE12 | – | 0.705 – 0.779 | – | – | 0.550 – 0.648 |
| DL1 – DL10 | – | – | 0.652 – 0.811 | – | 0.489 – 0.748 |
| DTSE1 – DTSE6 | – | – | – | 0.685 – 0.768 | 0.577 – 0.673 |

Note: Only primary loadings ≥ 0.40 are presented. All cross‑loadings were below 0.30. The factor structure perfectly matches the four intended constructs: Professional Identity (PI), Teaching Self‑Efficacy (TSE), Digital Literacy (DL), and Digital Teaching Self-Efficacy (DTSE).

### **Table S4. Standardized Factor Loadings and SMC for All Items (CFA)**

| Factor | Item | Standardized Loading | SMC (R²) |
| --- | --- | --- | --- |
| Digital Literacy (DL) | DL1 | 0.868 | 0.754 |
|  | DL2 | 0.809 | 0.655 |
|  | DL3 | 0.724 | 0.524 |
|  | DL4 | 0.654 | 0.428 |
|  | DL5 | 0.770 | 0.592 |
|  | DL6 | 0.754 | 0.569 |
|  | DL7 | 0.736 | 0.542 |
|  | DL8 | 0.699 | 0.489 |
|  | DL9 | 0.658 | 0.433 |
|  | DL10 | 0.782 | 0.612 |
| Professional Identity (PI) | PI1 | 0.803 | 0.644 |
|  | PI2 | 0.733 | 0.537 |
|  | PI3 | 0.709 | 0.502 |
|  | PI4 | 0.748 | 0.559 |
|  | PI5 | 0.724 | 0.523 |
|  | PI6 | 0.719 | 0.517 |
|  | PI7 | 0.725 | 0.526 |
|  | PI8 | 0.734 | 0.539 |
|  | PI9 | 0.720 | 0.519 |
|  | PI10 | 0.717 | 0.515 |
|  | PI11 | 0.694 | 0.481 |
|  | PI12 | 0.683 | 0.466 |
|  | PI13 | 0.677 | 0.458 |
|  | PI14 | 0.679 | 0.461 |
|  | PI15 | 0.707 | 0.499 |
|  | PI16 | 0.669 | 0.448 |
|  | PI17 | 0.677 | 0.458 |
| Teaching Self-Efficacy (TSE) | TSE1 | 0.784 | 0.615 |
|  | TSE2 | 0.756 | 0.571 |
|  | TSE3 | 0.714 | 0.510 |
|  | TSE4 | 0.753 | 0.566 |
|  | TSE5 | 0.725 | 0.526 |
|  | TSE6 | 0.740 | 0.548 |
|  | TSE7 | 0.759 | 0.575 |
|  | TSE8 | 0.781 | 0.610 |
|  | TSE9 | 0.716 | 0.512 |
|  | TSE10 | 0.768 | 0.590 |
|  | TSE11 | 0.749 | 0.562 |
|  | TSE12 | 0.783 | 0.613 |
| Digital Teaching Self-Efficacy Scale (DTSE) | DTSE1 | 0.765 | 0.585 |
|  | DTSE2 | 0.754 | 0.569 |
|  | DTSE3 | 0.710 | 0.505 |
|  | DTSE4 | 0.778 | 0.605 |
|  | DTSE5 | 0.767 | 0.588 |
|  | DTSE6 | 0.704 | 0.495 |

Note: All loadings are standardized and significant at p < 0.001. SMC = Squared Multiple Correlations (R²).

### **Table S5. Discriminant Validity: Fornell-Larcker Criterion**

| Factor | Digital Literacy | Professional Identity | Teaching Self-Efficacy | Digital Teaching Self-Efficacy |
| --- | --- | --- | --- | --- |
| Digital Literacy | 0.748 |  |  |  |
| Professional Identity | 0.413 | 0.713 |  |  |
| Teaching Self-Efficacy | 0.384 | 0.379 | 0.753 |  |
| Digital Teaching Self-Efficacy | 0.435 | 0.380 | 0.434 | 0.747 |

Note: Diagonal bold values are the square roots of the Average Variance Extracted (AVE). Off-diagonal values are the Pearson correlations among constructs. Discriminant validity is established when diagonal values (AVE) exceed all corresponding off-diagonal values (correlations) in the same row and column. All AVE square roots are greater than the inter-construct correlations (ranging from 0.379 to 0.435), supporting adequate discriminant validity.

### **Table S6. Heterotrait-Monotrait Ratio**

| Factor | Digital Literacy | Professional Identity | Teaching Self-Efficacy | Digital Teaching Self-Efficacy |
| --- | --- | --- | --- | --- |
| Digital Literacy | — |  |  |  |
| Professional Identity | 0.441 | — |  |  |
| Teaching Self-Efficacy | 0.411 | 0.402 | — |  |
| Digital Teaching Self-Efficacy | 0.481 | 0.417 | 0.477 | — |

Note: All HTMT values are below the recommended threshold of 0.85 (ranging from 0.402 to 0.481), confirming adequate discriminant validity among all four constructs.

### **Table S7. Model Fit Comparison: One-Factor vs. Four-Factor Model**

| Model | χ² | df | χ²/df | CFI | TLI | RMSEA | SRMR |
| --- | --- | --- | --- | --- | --- | --- | --- |
| Four-Factor Model (Proposed) | 1292.445 | 939 | 1.376 | 0.983 | 0.982 | 0.022 | 0.028 |
| One-Factor Model (Alternative) | 10358.703 | 945 | 10.962 | 0.536 | 0.514 | 0.113 | 0.132 |

Note: The one-factor model was estimated by constraining all 45 items to load onto a single latent factor. The four-factor model shows substantially better fit across all indices, with Δχ² = 9066.258, Δdf = 6, p < 0.001, supporting the distinctiveness of the four constructs.

### **Supplementary Table S8. Full Questionnaire Items for All Scales**

| Scale | Items | Response Format | Source |
| --- | --- | --- | --- |
| Digital Literacy Scale | 10 | 5‑point Likert | Ng (2012) |
| Professional Identity Scale | 17 | 7‑point Likert | Liu & Keating (2022) |
| Teaching Self‑Efficacy Scale (Short Form) | 12 | 9‑point scale | Tschannen-Moran and Hoy, 2001 |
| Digital Teaching Self-Efficacy Scale | 6 | 5‑point Likert | Kang, Yoo, & Kim (2024) |

The Digital Teaching Self-Efficacy Scale was adapted from the Teaching Execution subscale of the Scale of Competency of Digital Age Teaching (SCoDAT; Kang et al., 2024). The full questionnaire items are listed below for replication purposes. Copyright remains with the original authors and publishers.

**Scale 1: Digital Literacy Scale (Ng, 2012) – 10 items**

[1] I can use a range of digital tools effectively.

[2] I can evaluate the credibility of online information.

[3] I can create digital content for educational purposes.

[4] I can use digital tools to solve problems.

[5] I can adapt to new digital technologies quickly.

[6] I can use digital tools to collaborate with others.

[7] I can manage digital information efficiently.

[8] I can protect my privacy online.

[9] I can identify the ethical issues related to digital technology.

[10] I can use digital tools to support my learning.

**Scale 2: Professional Identity Scale (Liu and Keating, 2022) – 17 items**

[1] I am proud to be a PE teacher.

[2] I strongly identify with the PE teaching profession.

[3] I am willing to devote myself to PE teaching.

[4] I have a sense of belonging to the PE teacher community.

[5] I believe PE teaching is a valuable profession.

[6] I am confident in my ability to be an effective PE teacher.

[7] I feel that being a PE teacher is an important part of who I am.

[8] I actively seek opportunities to improve my PE teaching skills.

[9] I am committed to the professional development of PE teaching.

[10] I enjoy being a member of the PE teaching profession.

[11] I think PE teaching contributes positively to students' development.

[12] I feel valued as a PE teacher.

[13] I am enthusiastic about PE teaching.

[14] I would choose PE teaching again if I had the choice.

[15] I take pride in the achievements of my students.

[16] I feel a strong emotional attachment to PE teaching.

[17] I consider PE teaching as my lifelong career.

**Scale 3: Teaching Self-Efficacy Scale (Tschannen-Moran and Hoy, 2001) – 12 items**

[1] How much can you do to get through to the most difficult students?

[2] How much can you do to help your students think critically?

[3] How much can you do to control disruptive behavior in the classroom?

[4] How much can you do to motivate students who show low interest in schoolwork?

[5] How much can you do to get students to believe they can do well in schoolwork?

[6] How much can you do to help your students value learning?

[7] How much can you do to craft good questions for your students?

[8] How much can you do to use a variety of assessment strategies?

[9] How much can you do to provide alternative explanations when students are confused?

[10] How much can you do to implement alternative teaching strategies in your classroom?

[11] How much can you do to establish a classroom management system with each group of students?

[12] How much can you do to adjust your lessons to the proper level for individual students?

**Scale 4: Digital Teaching Self-Efficacy Scale (Kang, Yoo, and Kim, 2024) – 6 items**

[1] I can implement digital technology-integrated lessons effectively.

[2] I can manage classroom activities that involve student use of digital devices.

[3] I can facilitate student learning using digital tools during instruction.

[4] I can adjust my teaching in real-time based on student responses to technology-based activities.

[5] I can use digital tools to provide immediate feedback to students.

[6] I can maintain student engagement when using digital technology in the classroom.

**Response Scale Anchors Used in This Study**

Digital Literacy Scale: 5-point Likert (1 = strongly disagree, 5 = strongly agree)

Professional Identity Scale: 7-point Likert (1 = strongly disagree, 7 = strongly agree)

Teaching Self-Efficacy Scale: 9-point scale (1 = nothing, 9 = a great deal); Digital Teaching Self-Efficacy Scale: 5-point Likert (1 = strongly disagree, 5 = strongly agree)

## **Supplementary File S9:** Analysis Syntax Summary

| Analysis | Model | Specification | Output in Manuscript |
| --- | --- | --- | --- |
| Serial Mediation (Main) | PROCESS Model 6 | PROCESS y = DTSE_M /x = DL_M /m = PI_M TSE_M /model = 6 /cov = Gender Grade Intern /total = 1 /stand = 1 /contrast = 1 /boot = 5000 /seed = 12345. | Table 5, Table 6 |
| Parallel Mediation (Sensitivity) | PROCESS Model 4 | PROCESS y = DTSE_M /x = DL_M /m = PI_M TSE_M /model = 4 /cov = Gender Grade Intern /total = 1 /stand = 1 /contrast = 1 /boot = 5000 /seed = 12345. | Section 3.6 |
| Reverse-Ordered Serial Mediation (Sensitivity) | PROCESS Model 6 | PROCESS y = DTSE_M /x = DL_M /m = TSE_M PI_M /model = 6 /cov = Gender Grade Intern /total = 1 /stand = 1 /contrast = 1 /boot = 5000 /seed = 12345. | Section 3.6 |

## **Supplementary File S10:** AMOS CFA Model Specification

| Parameter | Specification |
| --- | --- |
| Software | IBM SPSS AMOS 31.0 |
| Estimation Method | Maximum Likelihood (ML) |
| Model Structure | 4 latent factors (Digital Literacy, Professional Identity, Teaching Self-Efficacy, Digital Teaching Self-Efficacy) |
| Item Assignment | DL1–DL10 → Digital Literacy; PI1–PI17 → Professional Identity; TSE1–TSE12 → Teaching Self-Efficacy; DTSE1–DTSE6 → Digital Teaching Self-Efficacy |
| Scale Identification | First item of each factor fixed to 1 (DL1, PI1, TSE1, DTSE1) |
| Factor Covariances | All four factors freely correlated |
| Residuals | Not correlated |
| Output in Manuscript | Table 3, Table 4, Supplementary Tables S4–S7 |

# **Supplementary File S11: Monte Carlo Simulation for Power Analysis**

### **Table S11-1. Population Model Parameters**

| Parameter | Description | Value |
| --- | --- | --- |
| a1 | PI ← DL | 0.413 |
| a2 | TSE ← DL | 0.273 |
| d21 | TSE ← PI | 0.266 |
| b1 | DTSE ← PI | 0.170 |
| b2 | DTSE ← TSE | 0.270 |
| cdash | DTSE ← DL (direct effect) | 0.263 |
| Res. Var. PI | Residual variance of PI | 0.829 |
| Res. Var. TSE | Residual variance of TSE | 0.792 |
| Res. Var. DTSE | Residual variance of DTSE | 0.704 |

Serial indirect effect: a1 × d21 × b2 = 0.413 × 0.266 × 0.270 = 0.0296

### **Table S11-2. Monte Carlo Simulation Power Estimates**

| Sample Size (N) | Replications | Statistical Power |
| --- | --- | --- |
| 100 | 1,000 | 0.236 |
| 200 | 1,000 | 0.849 |
| 300 | 1,000 | 1.000 |

Random seed = 12345. Power estimates represent the proportion of replications in which the serial indirect effect was statistically significant (p < 0.05).

### **Table S11-3. Mplus Model Results for IND_SERI**

| N | Average Estimate | Std. Dev. | S.E. Average | M.S.E. | 95% CI Coverage | Statistical Power |
| --- | --- | --- | --- | --- | --- | --- |
| 100 | 0.0289 | 0.0174 | 0.0169 | 0.000303 | 0.950 | 0.236 |
| 200 | 0.0293 | 0.0117 | 0.0118 | 0.000137 | 0.951 | 0.849 |
| 300 | 0.0297 | 0.0121 | 0.0119 | 0.000146 | 0.949 | 1.000 |

The M.S.E. values were calculated using the formula MSE = (Std. Dev.)^2 + (Average Estimate − 0.0296)^2, where 0.0296 is the true serial indirect effect (0.413 × 0.266 × 0.270). The 95% CI Coverage values (~0.95) indicate that the confidence intervals captured the true parameter in approximately 95% of the replications, as expected. These corrected M.S.E. and Coverage values replace the default Mplus-generated statistics, which were computed against an incorrect reference value due to software limitations in handling MODEL CONSTRAINT parameters. The power estimates (0.236, 0.849, 1.000 for N = 100, 200, 300) show that a sample size of approximately 200 participants is sufficient to achieve adequate power (≥ 0.80) for detecting the serial indirect effect, which is consistent with the sample size justification reported in the main manuscript (Section 2.2).

### **Mplus Syntax Used**

text

NOBS = 100 200 300; NREP = 1000; SEED = 12345; MODEL POPULATION: [DL*0]; DL@1; PI ON DL*0.413; PI@0.829; TSE ON PI*0.266 DL*0.273; TSE@0.792; DTSE ON TSE*0.270 PI*0.170 DL*0.263; DTSE@0.704; MODEL: DTSE ON TSE (b2); DTSE ON PI (b1); DTSE ON DL (cdash); TSE ON PI (d21); TSE ON DL (a2); PI ON DL (a1); MODEL CONSTRAINT: NEW(ind_serial); ind_serial = a1*d21*b2; OUTPUT: TECH9;

### The Monte Carlo simulations indicated that a sample size of approximately 200 participants is sufficient to achieve 0.80 power for detecting the serial indirect effect (power = 0.849). The actual sample (N = 776) substantially exceeds this threshold, confirming adequate statistical power for the study.

### Because these analyses relied on empirical path coefficients derived from the sample, they represent post hoc sensitivity checks rather than standalone a priori power analyses.

### **Supplementary Table S12. Complete Regression Coefficients Including Control Variables**

**Outcome Variable: Professional Identity**

| Predictor | b | SE | β | t | p | 95% CI |
| --- | --- | --- | --- | --- | --- | --- |
| Digital Literacy | 0.6042 | 0.0481 | 0.4133 | 12.5716 | 0.001 | [0.5099, 0.6986] |
| Gender | 0.0389 | 0.0896 | 0.0155 | 0.4338 | 0.6645 | [-0.1370, 0.2148] |
| Grade | -0.0176 | 0.0380 | -0.0152 | -0.4636 | 0.6430 | [-0.0923, 0.0570] |
| Internship | -0.0135 | 0.0939 | -0.0051 | -0.1436 | 0.8858 | [-0.1978, 0.1709] |

R² = 0.171, F(4, 771) = 39.68, p < 0.001

**Outcome Variable: Teaching Self-Efficacy**

| Predictor | b | SE | β | t | p | 95% CI |
| --- | --- | --- | --- | --- | --- | --- |
| Digital Literacy | 0.4717 | 0.0611 | 0.2726 | 7.7227 | 0.001 | [0.3518, 0.5916] |
| Professional Identity | 0.3152 | 0.0417 | 0.2664 | 7.5609 | 0.001 | [0.2334, 0.3971] |
| Gender | -0.1205 | 0.1038 | -0.0406 | -1.1611 | 0.2459 | [-0.3242, 0.0832] |
| Grade | -0.0021 | 0.0441 | -0.0015 | -0.0472 | 0.9623 | [-0.0886, 0.0844] |
| Internship | 0.0250 | 0.1087 | 0.0080 | 0.2296 | 0.8184 | [-0.1885, 0.2384] |

R² = 0.208, F(5, 770) = 40.31, p < 0.001

**Outcome Variable: Digital Teaching Self-Efficacy**

| Predictor | b | SE | β | t | p | 95% CI |
| --- | --- | --- | --- | --- | --- | --- |
| Digital Literacy | 0.2493 | 0.0328 | 0.2629 | 7.6075 | 0.001 | [0.1849, 0.3136] |
| Professional Identity | 0.1103 | 0.0223 | 0.1700 | 4.9374 | < 0.001 | [0.0664, 0.1541] |
| Teaching Self-Efficacy | 0.1481 | 0.0186 | 0.2703 | 7.9540 | 0.001 | [0.1116, 0.1847] |
| Gender | 0.0198 | 0.0537 | 0.0122 | 0.3680 | 0.7130 | [-0.0856, 0.1251] |
| Grade | -0.0018 | 0.0228 | -0.0024 | -0.0776 | 0.9382 | [-0.0465, 0.0429] |
| Internship | 0.0291 | 0.0562 | 0.0171 | 0.5173 | 0.6051 | [-0.0812, 0.1394] |

R² = 0.296, F(6, 769) = 53.95, p < 0.001

b = unstandardized coefficient; SE = standard error; β = standardized coefficient; CI = confidence interval.
